# Nocodazole Treatment Decreases Expression of Pluripotency Markers Nanog and Oct4 in Human Embryonic Stem Cells

**DOI:** 10.1371/journal.pone.0019114

**Published:** 2011-04-29

**Authors:** Ade Kallas, Martin Pook, Martti Maimets, Külli Zimmermann, Toivo Maimets

**Affiliations:** Institute of Molecular and Cell Biology, University of Tartu, Tartu, Estonia; University of Bristol, United Kingdom

## Abstract

Nocodazole is a known destabiliser of microtubule dynamics and arrests cell-cycle at the G2/M phase. In the context of the human embryonic stem cell (hESC) it is important to understand how this arrest influences the pluripotency of cells. Here we report for the first time the changes in the expression of transcription markers Nanog and Oct4 as well as SSEA-3 and SSEA-4 in human embryonic cells after their treatment with nocodazole. Multivariate permeabilised-cell flow cytometry was applied for characterising the expression of Nanog and Oct4 during different cell cycle phases. Among untreated hESC we detected Nanog-expressing cells, which also expressed Oct4, SSEA-3 and SSEA-4. We also found another population expressing SSEA-4, but without Nanog, Oct4 and SSEA-3 expression. Nocodazole treatment resulted in a decrease of cell population positive for all four markers Nanog, Oct4, SSEA-3, SSEA-4. Nocodazole-mediated cell-cycle arrest was accompanied by higher rate of apoptosis and upregulation of p53. Twenty-four hours after the release from nocodazole block, the cell cycle of hESC normalised, but no increase in the expression of transcription markers Nanog and Oct4 was detected. In addition, the presence of ROCK-2 inhibitor Y-27632 in the medium had no effect on increasing the expression of pluripotency markers Nanog and Oct4 or decreasing apoptosis or the level of p53. The expression of SSEA-3 and SSEA-4 increased in Nanog-positive cells after wash-out of nocodazole in the presence and in the absence of Y-27632. Our data show that in hESC nocodazole reversible blocks cell cycle, which is accompanied by irreversible loss of expression of pluripotency markers Nanog and Oct4.

## Introduction

Human embryonic stem cells (hESC) are characterised by pluripotency, unlimited proliferative growth potential and a short cell division cycle due to an abbreviated G1 phase. A distinct set of transcription factors (Sox2, Oct4, Nanog) are responsible for maintaining cell pluripotency and undifferentiated phenotypes of cells. Suppression of Oct4 expression in hESC leads to loss of pluripotency and induces expression of differentiation markers specific for the trophectoderm [1], [2] or endoderm [3]. Transgene-mediated overexpression of Oct4 triggers differentiation of embryonic stem cells into endodermal or mesodermal structures [4], [5]. Experimental knockdown of another transcription factor, Nanog, leads to hESC differentiation towards embryonic or extraembryonic lineages, depending on the experimental conditions and cell line-intrinsic determinants [6], [7], [8]. Conversely to the effect of Oct4 overexpression, the overexpression of Nanog promotes self-renewal of hESC in the absence of any feeders [9]. Sox2 forms a dimeric complex with Oct4 and mediates transcription of several stem-cell specific genes, including their own promoter and that of Nanog [10], [11]. Transcription factors Oct4 and Sox2 are also involved in reciprocal regulation of each other's expression [12]. Despite the effectiveness of the network of transcription factors in promoting and maintaining pluripotency, their mode of action remains unclear.

Microtubule-targeted agents like taxol, vinca alkaloids, colcemid and nocodazole have been studied extensively in diverse types of cell lines, including hESC cultures. These agents interfere with microtubule polymerisation and cause arrest in the G2/M phase of the cell cycle. Taxol binds to β-tubulin and stabilises microtubules by making them rigid and less dynamic [13]. The outcome of taxol treatment depends on the concentration used and differs in different cell lines [14], [15]. Nocodazole acts as a microtubule destabiliser with the opposite effect of taxol. Still, it is effective in disturbing microtubule dynamics and arresting cell cycle progression at mitosis. Nocodazole has been used to arrest hESC cells in the G2/M phase of the cell cycle. However, there is no information regarding the effect of nocodazole on the pluripotency markers Nanog and Oct4.

hESC lines are sensitive and any change of important ingredients in the basic culture protocol or routine manipulation, such as passaging and cryopreservation, could lead to various degrees of differentiation and loss of pluripotency [16], [17]. The p160-Rho-associated coiled-coil kinase 2 (ROCK2) inhibitor Y-27632 is a promising agent in hESC culture methods, since it improves cell proliferation [18], [19], [20] and recovery of frozen-thawed variant pluripotent stem cell types, including hESC and induced pluripotent stem cells [21], [22], [23]. It is also effective in karyotypically normal hESC and variant hESC without any changes in cell cycle progression or morphology [24]. ROCK-2 inhibitor Y-27632 increases the expression of genes of stemness-related integrins (αV, α6 and β1), which in turn increase ECM-cell interaction [25]. Recently the ability of Y-27632 to inhibit myosin light chain phosphorylation has been shown to be responsible for increased cloning efficiency of hESC [26].

In this study we investigated the effect of nocodazole on hESC pluripotency evaluated by the expression of markers Oct4, Nanog, SSEA-3 and SSEA-4. We applied flow cytometry to analyse distinct populations of cells in the different cell cycle phases. Using this approach, we now report that the nocodazole treatment of hESC results in loss of pluripotency markers Oct4 and Nanog. After being released from nocodazole-caused arrest, the expression of these pluripotency markers remained at the same low level, but the cells were capable of continuing cell growth and up-regulating the expression of SSEA-3 and SSEA-4 in the Nanog-expressing cell population in the absence or presence of Y-27632. These results show, that in hESC nocodazole reversible blocks cell cycle, which is accompanied by irreversible loss of expression of pluripotency markers Nanog and Oct4.

## Materials and Methods

### Ethics statement

This study was conducted using a commercially available human embryonic stem cell line (WA09, National Stem Cell Bank, Madison, WI, USA); no *in vivo* experiments in animals or humans were performed and therefore approval from the ethics committee was not necessary. Approval to isolate mouse embryonic fibroblasts from the embryos of CD-1 mice (Animal Facility of the Institute of Cell and Molecular Biology, Tartu, Estonia) was obtained from the Committee for Experiments on Laboratory Animals, Estonian Ministry of Agriculture.

### Cell culture

Human ES cell line H9 (WA09, National Stem Cell Bank, Madison, WI, USA) was cultured on Matrigel® (BD Biosciences, San Jose, CA, USA) coated plates by using the mTeSR™1 maintenance medium (STEMCELL Technologies Inc, Vancouver, Canada) according to the manufacturer's specifications. The medium was changed daily. After 7 days of growth colonies were detached mechanically from the feeder cells with a micropipette tip. After breaking the colonies by gentle pipetting, individual hESC clumps were plated onto fresh Matrigel® coated plates.

### Antibodies and reagents

Alexa-488-labelled anti-H3 antibodies specific for Ser10 phosphorylation, phospho-Ser28-specific anti-H3 antibodies, Alexa-488-labelled anti-p53 antibodies and anti-cleaved caspase-3 antibodies were obtained from Cell Signaling Technology (Beverly, MA). Alexa 647-labelled anti-Oct4 antibodies and their isotype control were obtained from e-Bioscience. PE-labelled anti-Nanog antibodies, Alexa 647-labelled anti-Gata 4 antibodies, Alexa-647-labelled anti-SSEA-4 antibodies, Alexa-488-labelled anti-SSEA-3 antibodies and their isotype controls were purchased from BD Biosciences. Anti-p21 ^WAF1/CIP1^ antibodies and another anti-Oct4 antibodies were purchased from Santa Cruz (Santa Cruz Biotechnology, San Diego, CA, USA). Nocodazole (Sigma-Aldrich Chemicals, St. Louis, MO, USA) was dissolved and diluted in DMSO (Sigma-Aldrich Chemicals). ROCK-2 inhibitor Y-27632 was purchased from Tocris Bioscience (Bristol, UK).

### Multivariate permeabilised-cell flow cytometry and cell cycle analysis

Cell cultures were expanded for 5–6 days on 6-well plates and treated with various concentrations of nocodazole or DMSO only as a control, or grown without treatment at 37°C in 5% CO_2_ humidified atmosphere. After harvesting with 0.05% trypsin-EDTA solution (PAA Laboratories, Linz, Austria) and washing with PBS, single hES cell suspensions were fixed by using 1.6% paraformaldehyde (PFA, Sigma-Aldrich) for 10 min at RT as described for detection of intracellular phosphoproteins [27], [28]. After that cells were washed and stained using a permeabilisation buffer (Foxp3 Staining Buffer Set, e-Biosciences). Cells were blocked using 2% goat serum in a permeabilisation buffer (15 min at RT) and stained with appropriate antibodies or their isotype controls for 30 min at RT. For cell cycle analysis the cells were stained with DAPI (Cystain DNA, Partec GmbH, Münster, Germany). Flow cytometry data were acquired with FACSAria using FACSDiva software (BD Biosciences). In some experiments after trypsinisation, cells were first stained with anti-SSEA-3 and anti-SSEA-4 antibodies and then fixed and permeabilised for further staining with anti-Nanog antibodies.

The number of apoptotic cells was estimated after incubation of an aliquot of collected living cells with PE-labelled Annexin-V (BD Pharmingen) and with 7-AAD. Co-expression of Nanog and Oct4 in apoptotic cells was measured by using anti-cleaved caspase-3 antibodies followed with chicken anti-rabbit IgG antibodies labelled with Alexa Fluor 488.

Cell permeabilisation, fixation, staining and data acquisition for all samples were done on the same day.

### Western blot analysis

Protein samples were electrophoresed on SDS polyacrylamide gel (10%) and were transblotted (MiniTransblot Cell, Bio-Rad, Hercules, CA) onto a polyvinylidene difluoride membrane (Millipore, Billerica, MA). The membranes were probed with rabbit anti-Nanog antibodies (Aviva Systems Biology, San Diego, CA, USA), mouse anti-Oct4 antibodies (Santa Cruz Biotechnology) and followed by horseradish peroxidase-conjugated goat anti-rabbit or goat anti-mouse secondary antibodies. Expression of p53 was detected by incubating the membrane with rabbit anti-p53 antibodies (Cell Signaling Technology). Mouse anti-beta-actin antibodies (Abcam, Cambridge, MA, USA) were used for loading control. Binding of antibodies was detected with ECL reagent (Western Lightning Plus-ECL, PerkinElmer Inc, Waltham, MA, USA) and exposing the blots on X-ray films (Amersham Biosciences).

### Statistical analysis

A two-tailed paired t-test with a confidence interval of 95% was used to analyse the data with GraphPad Prism 4 software. *p* value less than 0.05 was considered significant. All results are presented as mean ± standard error. The distribution of cells in each phase of the cell cycle was calculated using ModFit software.

## Results

### The effect of various concentrations of nocodazole on hESC

**Figure 1:**
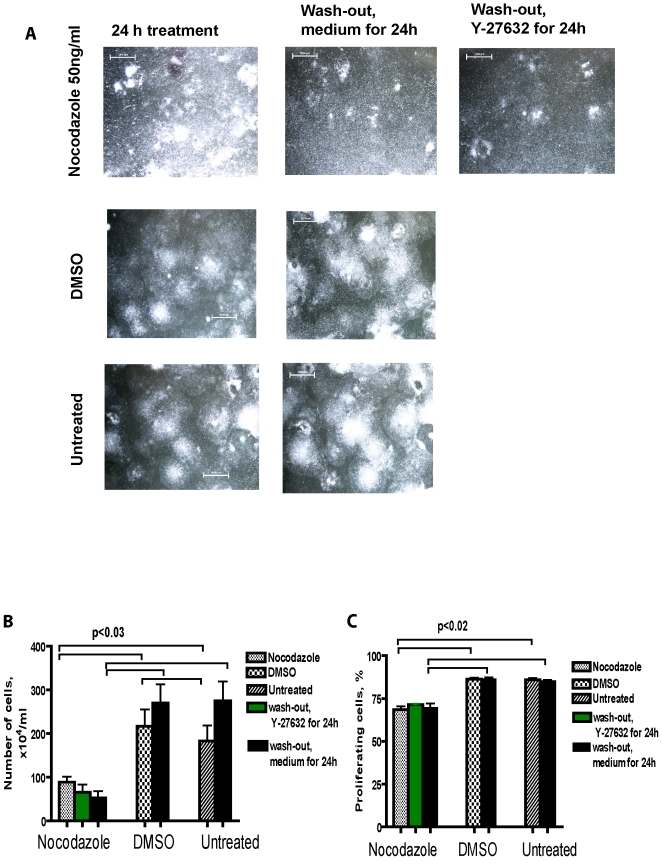
Effect of nocodazole on hESC morphology. (A) Cells were treated with nocodazole 50 ng/ml, or with DMSO for 24 h or left untreated. After nocodazole treatment, cells were washed with medium and grown in the presence or in the absence of Y-27632 (20 µM) for further 24 h. (B) Number of adherent cells after nocodazole treatment compared to DMSO-treated cells and untreated cells. Scale bar = 1000 µm (C) Proliferating cells of adherent cells estimated by using DAPI staining.

To find out the most suitable concentration of nocodazole for further experiments we first performed a dose-response experiment. 50, 100 and 200 ng/ml nocodazole was added to the hESC for 24 h. Then the cells were harvested and after staining with appropriate antibodies analysed by flow cytometry for cell cycle distribution and expression of pluripotency markers Nanog and Oct4. Nocodazole treatment caused morphological changes in colonies: only multilayer dense areas of colonies survived. We noticed progressive cell detachment and cell death (Figure 1). DMSO-treated and untreated cell colonies expressed well-defined borders and the colonies were so dense that individual cells could not be distinguished.

The number of adherent cells decreased significantly after nocodazole treatment compared to DMSO-treated or untreated cells. Within the adherent cell population we detected a decrease in the proliferating cell population caused by nocodazole treatment (Figure 1 BC).

**Figure 2:**
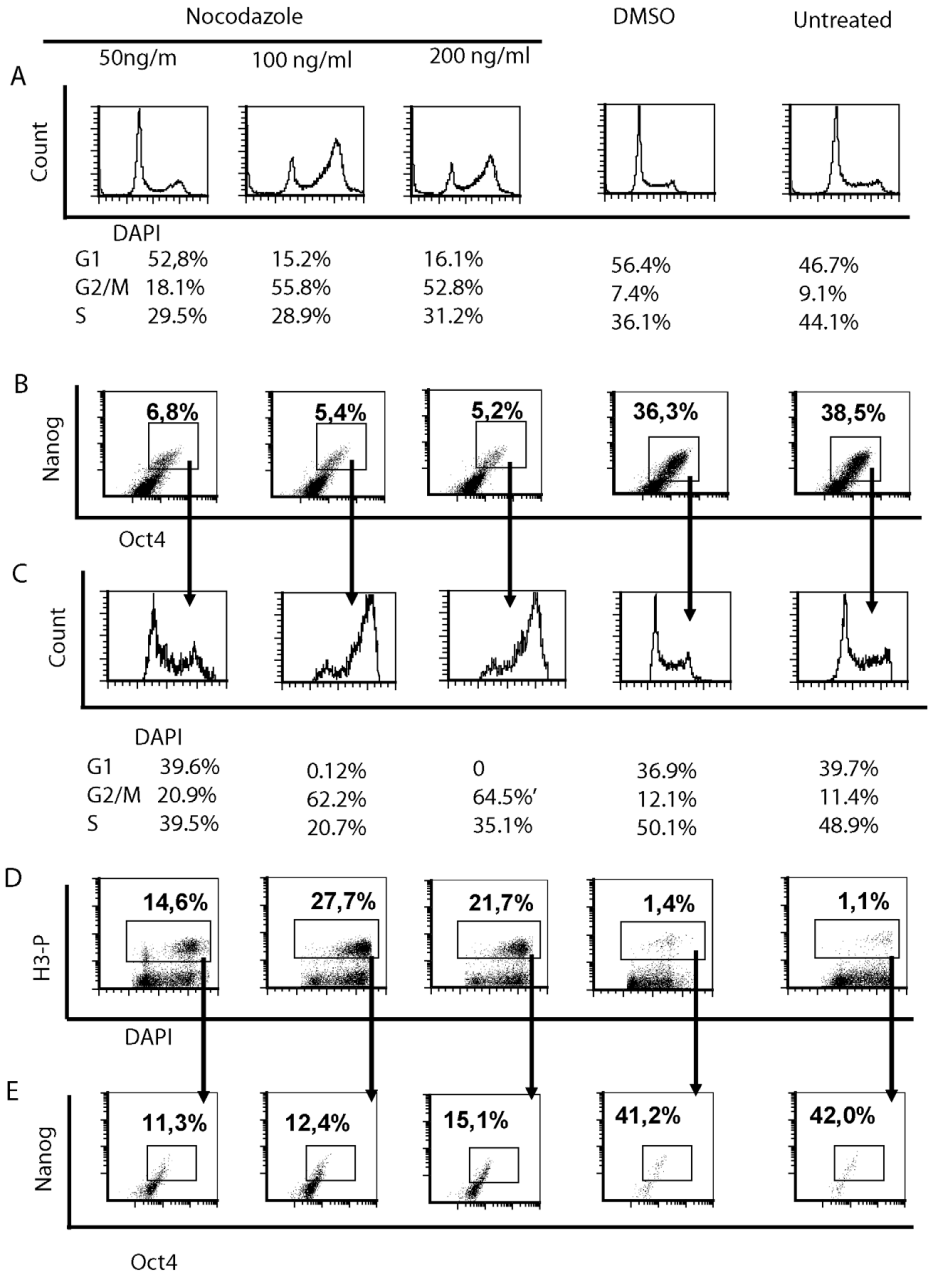
Effect of various concentrations of nocodazole on pluripotent hESC cells. H9 hESC were exposed to different concentrations of nocodazole or DMSO for 24 h or left untreated. Fixed and permeabilised cells were stained with anti-H3-P-Ser10 (Alexa Fluor 488 conjugate), anti-Nanog (PE), anti-Oct4 (Alexa Fluor 647) antibodies and with DAPI. For analysis cellular debris and doublets were excluded. (A) Cell cycle profile determined by DAPI staining. Number of cells in each cell cycle phase as estimated by using ModFit software. (B) Flow cytometric analysis of Nanog and Oct4 co-expressing cells and (C) their distribution in different cell cycle phases (stained with DAPI). (D) Phosphorylation of H3 at Ser10 in cells of different cell cycle phases and (E) expression of Nanog and Oct4 in these cells, which possessed phosphorylated H3. Results are representative of two independent experiments.

**Figure 3:**
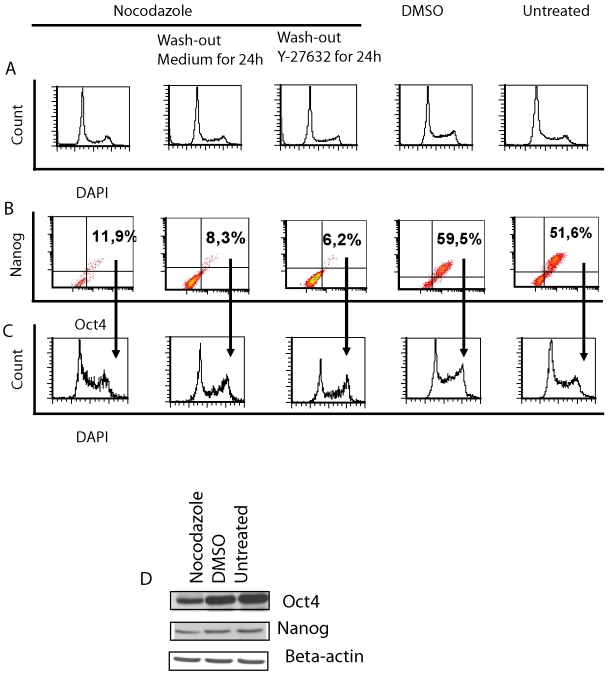
Characterisation of nocodazole-treated and released hES cells. Cells were arrested with 50 ng/ml nocodazole followed by washing and expansion with fresh medium for further 24 h in the presence or in the absence of Y-27632. (A) Representative histograms of cell cycle distribution. (B) FACS analysis of pluripotency markers Nanog and Oct4 (results shown as density plots) and (C) distribution of these Nanog^pos^Oct^pos^ cells in cell cycle phases. (D) Expression of Nanog and Oct4 in cell lysates detected by using Western blot assay. Beta-actin was used as loading control. Results are representative of four independent experiments.

The distribution of cells in cell cycle phases changed after nocodazole treatment. Whereas the untreated and DMSO-treated cells were mostly in the S or in G1 phases, then after nocodazole treatment we found fewer cells in the S phase and increased number of cells in the G2/M phase (Figure 2A). Higher concentrations of nocodazole resulted in a higher number of cells in the G2/M phase and fewer cells in the G1 phase, according to the cell cycle analysis with ModFit software (Figure 2A). The expression of pluripotency markers Nanog and Oct4 in these cells was evaluated by using simultaneous staining with appropriate antibodies. We could detect a population of cells, which was Nanog and Oct4 double-positive, but no single positive cell population (Figure 3B). The expression of Nanog correlated with the expression of Oct4. Nocodazole treatment caused a decrease in the fraction of cells co-expressing Nanog and Oct4. When the distribution of these double-positive cells in each cell cycle phase was estimated, then a higher number of cells was found in the G2/M phase (Figure 2C).

To find out whether nocodazole caused cell arrest in mitosis, we evaluated the phosphorylation level of histone 3 (H3) at Ser10. Phosphorylation of H3 was significantly increased after treatment with nocodazole, compared to the treatment with DMSO as a control (Figure 2D). Higher doses of nocodazole resulted in higher phosphorylation levels of H3 at Ser 10. Cells with phosphorylated H3 were in the G2/M phase of the cell cycle. When the lowest concentration of nocodazole was used, we also found some cells with phosphorylated H3 in the G1 phase (Figure 2D). There was a good correlation between the phosphorylation levels of H3 and the number of cells in the G2/M phase (Spearman non-parametric correlation r = 0.895; p<0.0001). We further evaluated whether the cells with phosphorylated H3 expressed pluripotency markers Nanog and Oct4. Nocodazole treatment caused an increase in the phosphorylation level of H3, but co-expression of Nanog and Oct4 in these cells decreased when compared to DMSO-treated or untreated cells (Figure 2E). In further experiments we used a low dose (50 ng/ml) of nocodazole for several reasons: (a) it had the lowest effect on cell survival; (b) the number of cells in the G2/M phase increased and this was accompanied with an elevated level of H3 phosphorylation; (c) cells expressing both Nanog and Oct4 were also detectable.

### Nocodazole has a reversible effect on cell cycle arrest, but not on pluripotency markers Nanog and Oct4

Since the number of attached cells decreased significantly after nocodazole treatment, we then investigated whether the remaining cells could proliferate after the release from nocodazole and upregulate the expression of pluripotency markers Nanog and Oct4. After 24 h of the nocodazole block the cells were intensively washed with a growth medium and cultured for a further 24 h with a fresh medium lacking nocodazole. To some cells a selective ROCK-2 inhibitor (Y-27632), which improves survival of hESC upon single-cell dissociation and enhances recovery from cryopreservation, was added. The short time period was chosen in order to capture the initial changes in colony composition. After nocodazole wash-out, the survival of cells decreased further, but we detected no changes in the number of proliferating cells (Figure 1BC). The morphologies of colonies grown in the presence or in the absence of Y-27632 after nocodazole treatment were compared to the colonies grown without any treatment. More flattened morphology was noticed in the presence of Y-27632 than in the absence of that at 24 h (Figure 1). The formation of small colonies from dissociated cells was noticed in the presence of Y-27632 in some plates.

**Figure 4:**
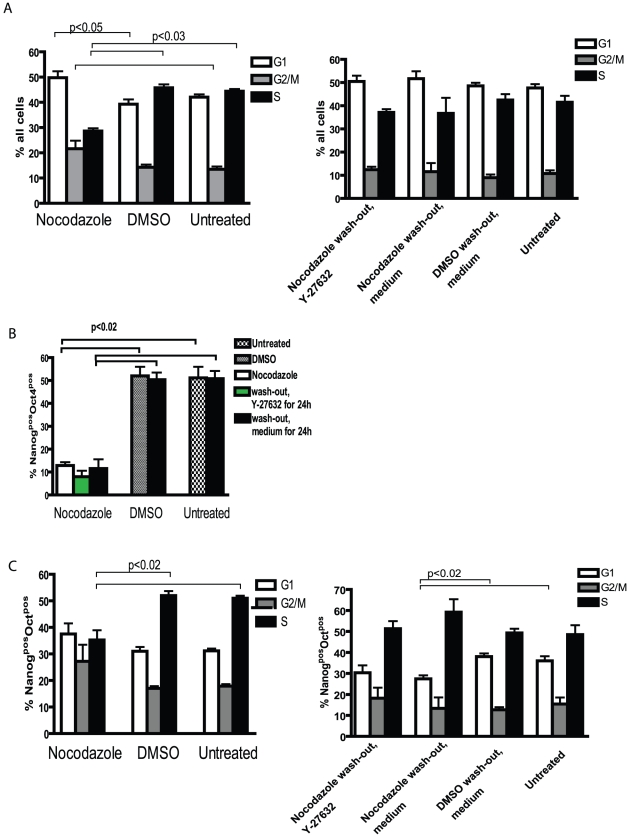
Cell cycle analysis of nocodazole-treated and released hESC. H9 hESC were exposed to nocodazole as described in Figure 3. (A) Distribution of all cells in different cell cycle phases analysed by using ModFit software. (B) Summarising bar graph of flow cytometric analysis of Nanog^pos^Oct4^pos^ cells and (C) their distribution in cell cycle phases. Results are shown as mean ± SEM of four independent experiments.

**Figure 5:**
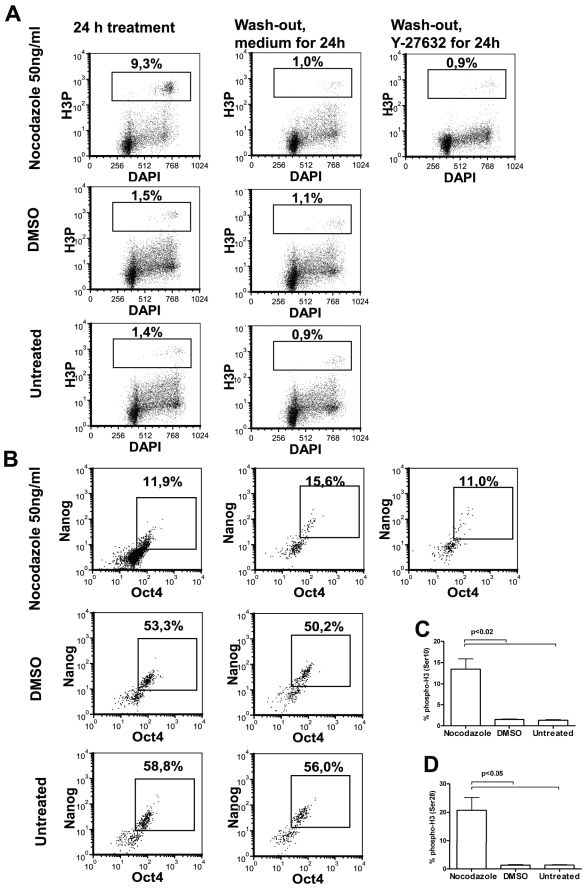
Histone 3 phosphorylation in nocodazole-treated and released hESC. hESC were exposed to nocodazole as described in Figure 3. (A) Phosphorylation of H3 at Ser 10 in the G2/M phase of cell cycle (density plots). (B) Expression of Nanog and Oct4 in cells with phosphorylated H3 as gated out in section A. (C) Phosphorylation of histone 3 at Ser10 or (D) Ser28. Results are shown as mean ± SEM of four independent experiments.

Cell cycle profile returned to normal after 24 h of nocodazole wash-out (Figure 4A): the number of cells in the S phase increased and in the G2/M phase decreased and was comparable with DMSO-treated or untreated cells (Figure 4A). After being released from nocodazole arrest, the fraction of Nanog and Oct4 co-expressing cells further decreased slightly (Figure 4B). A significant decrease in the distribution of these double-positive cells in the G1 phase of the cell cycle was detected after removal of nocodazole (Figure 4C). In comparison, DMSO treatment and subsequent washout had no effect on the number of Nanog^pos^Oct4^pos^ cells (Figure 4B) and their distribution in cell cycle phases (Figure 4C). Phosphorylation of H3 after removal of nocodazole decreased to the level comparable with DMSO-treated cells and untreated cells (Figure 5A). In nocodazole treated cells we detected a significant decrease in a fraction of cells with phosphorylated H3 co-expressing Nanog and Oct4 compared to DMSO-treatment or untreated cells (Figure 5B). In addition, we evaluated the phosphorylation of H3 at Ser28, which was comparable to the results of phosphorylation of H3 at Ser10 (Figure 5CD). The addition of Y-27632 to DMSO-treated or untreated cells had no significant effect on the expression of pluripotency markers Nanog and Oct4, cell cycle profile or expression of undifferentiation markers SSEA-3 and SSEA-4 (Figure S1).

### Expression of SSEA-3 and SSEA-4 in hESC treated with nocodazole

**Figure 6:**
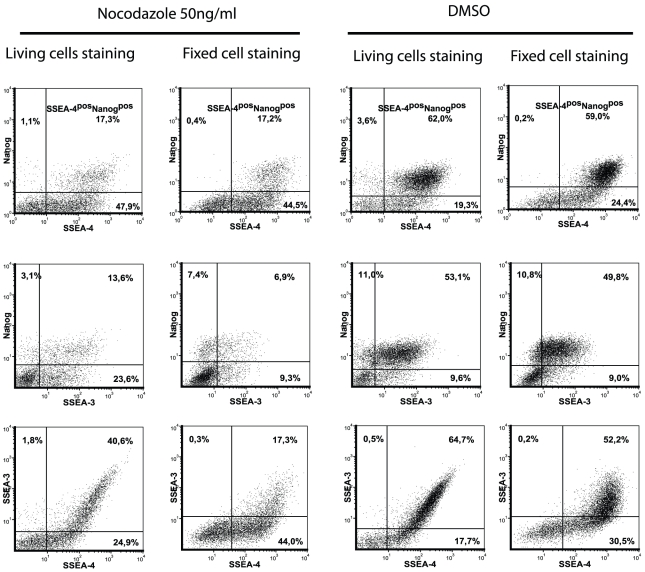
Detection of SSEA-3 and SSEA-4 on fixed or living hESC. hESC were first stained with anti-SSEA-3 (Alexa Fluor 488), anti-SSEA-4 (Alexa Fluor 647) antibodies and thereafter cells were fixed, permeabilised and stained with anti-Nanog (PE) antibodies (living cells). Alternatively, the cells were stained after fixation with all three antibodies (fixed cells). For analysis cellular debris and doublets were excluded and DAPI-positive cells were selected. Results are shown as density plots.

We next investigated whether nocodazole treatment has any effect on the glycosphingolipids on the cell surface. Thus the expression of SSEA-3 and SSEA-4 was studied using two different cell-staining procedures for analysis on a flow cytometer. First, the cells were stained with antibodies specific to stage-specific embryonic antigens SSEA-3 and SSEA-4 (monoclonal antibodies MC631 and MC813-70, respectively), thereafter fixed, permeabilised and stained with Nanog-specific antibodies. Another approach was to fix cells first with 1.6% PFA and then cells were co-stained with antibodies specific to SSEA-3, SSEA-4 and Nanog. Both staining methods revealed two main cell populations: SSEA-4^pos^Nanog^pos^ cells and SSEA-4^pos^Nanog^neg^ cells (Figure 6). These cells, which expressed SSEA-4 at a high level, were also positive for Nanog expression, while the cells with a low level of SSEA-4 expression had no detectable level of Nanog (Figure 6). The majority of SSEA-3 expressing cells were positive for Nanog expression in nocodazole-treated, DMSO-treated or untreated cells. There was a positive correlation between expression of SSEA-3 and SSEA-4: all the cells with high expression of SSEA-4 also expressed SSEA-3, which is in agreement with the previous finding that both of these antibodies recognise a common epitope (GL7) in hESC glycosphingolipids. In addition, SSEA-3 antibodies bind to another epitope GL5, but SSEA-4 antibodies cross-react to a different extent with other glycolipids and recognise another epitope common in glycoproteins [29]. This could explain wider and continuous distribution of SSEA-4 positive cells. SSEA-3 antibodies had a higher ability to bind to living cells (recognising the internal structure of GL7 epitope), but SSEA-4-specific antibodies to fixed cells (recognise the terminal structure of GL7 epitope) [29]. Unspecific binding of both SSEA-3 and SSEA-4 antibodies was lower, when living (non-fixed) cells were stained. Fixation of cells even with 1.6% PFA considerably increased non-specific binding with antibodies conjugated with Alexa Fluor 647. It was also noted that non-specific binding in nocodazole-treated cells was higher than in DMSO-treated or untreated cells. Since the tendencies by using both methods were similar, we applied the staining of fixed cells in the following experiments.

**Figure 7:**
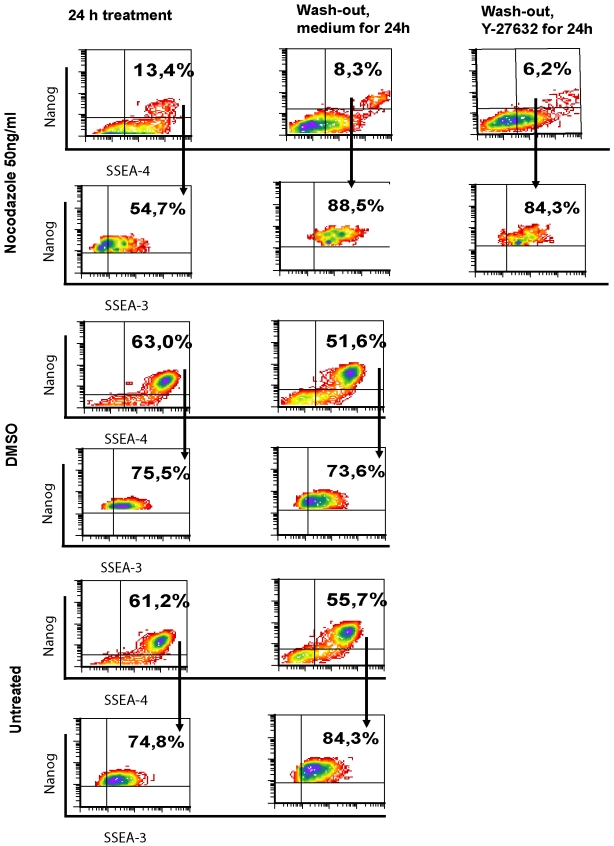
SSEA-3 and SSEA-4 expression in Nanog-positive cells. hESC were exposed to nocodazole as described in Figure 3. Cells were fixed with 1.6% PFA and stained with anti-SSEA-3 (Alexa Fluor 488), anti-Nanog (PE), anti-SSEA-4 (Alexa Fluor 647) antibodies and DAPI. Cells co-expressing SSEA-4 and Nanog were characterised for expression of SSEA-3. Results are shown as density plots and are representative of four independent experiments.

SSEA-3 and SSEA-4 expressions were studied in cells treated with nocodazole and after its removal in the presence and absence of Y-27632. As the number of Nanog^pos^ cells decreased after nocodazole treatment and wash-out, we could detect a slightly lower number of SSEA-4^pos^ Nanog^pos^ cells, but a higher number of SSEA-4^pos^Nanog^neg^ cells after treatment with nocodazole compared to DMSO treatment (Figure 7, 8BC). When the SSEA-4^pos^ Nanog^pos^ cell population was selected and investigated for SSEA-3 expression, we found that the majority of cells were SSEA-3 positive (Figure 7, 8D). Wider distribution of SSEA-4 and SSEA-3 expression was noticed after 24 h treatment in the presence of Y-27632 compared to its absence in nocodazole-released cells. The removal of nocodazole resulted in an increase of the mean fluorescence intensity of SSEA-4 and SSEA-3 in SSEA-4^pos^Nanog^pos^ and SSEA-3^pos^Nanog^pos^ cell populations, respectively, indicating up-regulation of SSEA-4 and SSEA-3 expression (e.g. epitope GL7 in globo-series glycolipids) in these Nanog-expressing cells (Figure 7, 8BD). This effect was detectable after 24 h growth in a fresh medium and the expression also remained elevated at 48 h (data not shown). In some experiments, nocodazole treatment for 16 h was used, which was not different from treatment for 24 h. Thus, the expression of the Nanog pluripotency marker decreased as a result of nocodazole treatment, whereas the cells remained undifferentiated since the SSEA-4-specific antibodies detected globo-series glycolipids on the cell surface.

**Figure 8:**
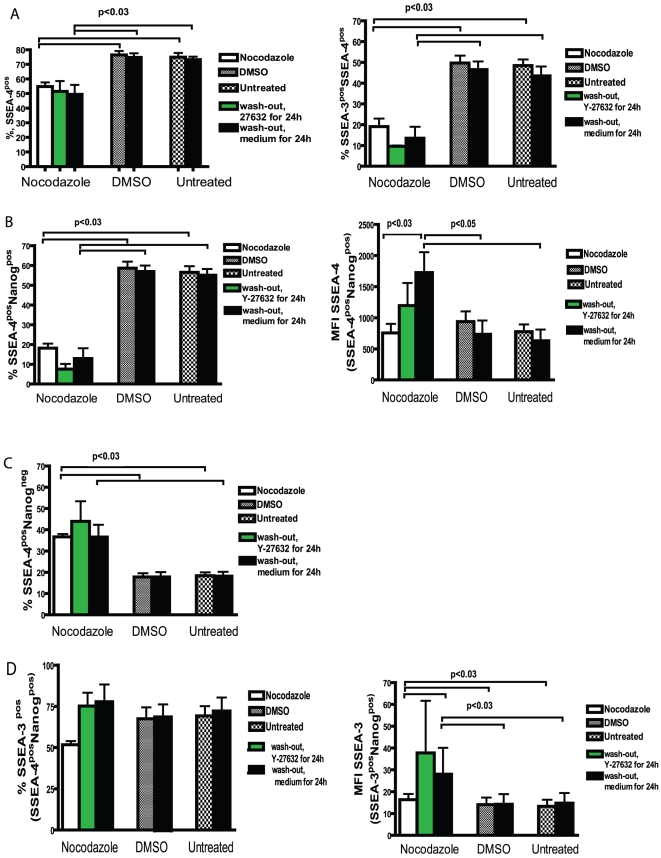
Characterisation of SSEA-4, SSEA-3 and Nanog expression in hESC released from nocodazole treatment. Summarising bar graphs of flow cytometric analysis of cells stained as described in Figure 7. (A) Number of SSEA-4 expressing cells and SSEA-4^pos^SSEA-3^pos^ cells after treatment with nocodazole and following release from nocodazole arrest in presence or absence of Y-27632. (B) Number of Nanog expressing and SSEA-4-positive cells. Mean fluorescence intensity (MFI) of SSEA-4 expression of SSEA-4^pos^Nanog^pos^ population. (C) Number of SSEA-4 expressing and Nanog-negative cells. (D) Expression of SSEA-3 in SSEA-4^pos^Nanog^pos^ cell population. MFI of SSEA-3 expression of SSEA-3^pos^Nanog^pos^ population. Results are shown as mean ± SEM of four independent experiments.

### Expression of Gata-4 in nocodazole-treated hESC

**Figure 9:**
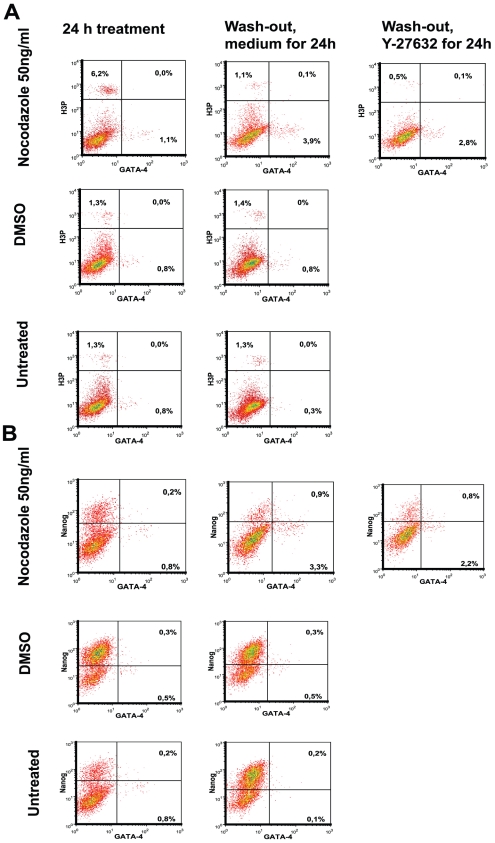
Expression of GATA-4 in hESC. H9 hESC were treated as described in Figure 3. Fixed and permeabilised cells were stained with anti-H3-P-Ser10 (Alexa Fluor 488 conjugate), anti-Nanog (PE), anti-GATA-4 (Alexa Fluor 647) antibodies and DAPI. (A) Expression of GATA-4 and phosphorylated H3 at Ser10 in hESC. (B) Number of Nanog and GATA-4 expressing hESC. Results are shown as density plots.

We further asked whether the cells with non-detectable globo-series glycolipids on the cell surface (SSEA-4 negative cell population) were more prone to differentiation. The number of differentiated cells was estimated by using antibodies specific to Gata-4, since cells with a low level of Nanog expression have reported to express Gata-4 [30]. The increased level of Gata-4 transcripts has been detected in the hESC upon Oct4 knockdown [3]. The number of cells producing Gata-4 was rather low, ranging from 0.4% to 3.9% (Figure 9), but increased Gata-4 expression was noticed in nocodazole-treated and released cells, when compared to DMSO-treated (and released) cells. To investigate whether these Gata-4 expressing cells were in the G2/M phase, we used co-staining with Gata-4 antibodies and phosphor-H3-specific antibodies. hESC with Gata-4 expression had no phosphorylated H3 and this result did not depend on the method of treatment (Figure 9A). A similar low level of Nanog expression was noticed in Gata-4-positive cells in all studied cell sets (Figure 9B).

### Nocodazole induces apoptosis in hESC

**Figure 10:**
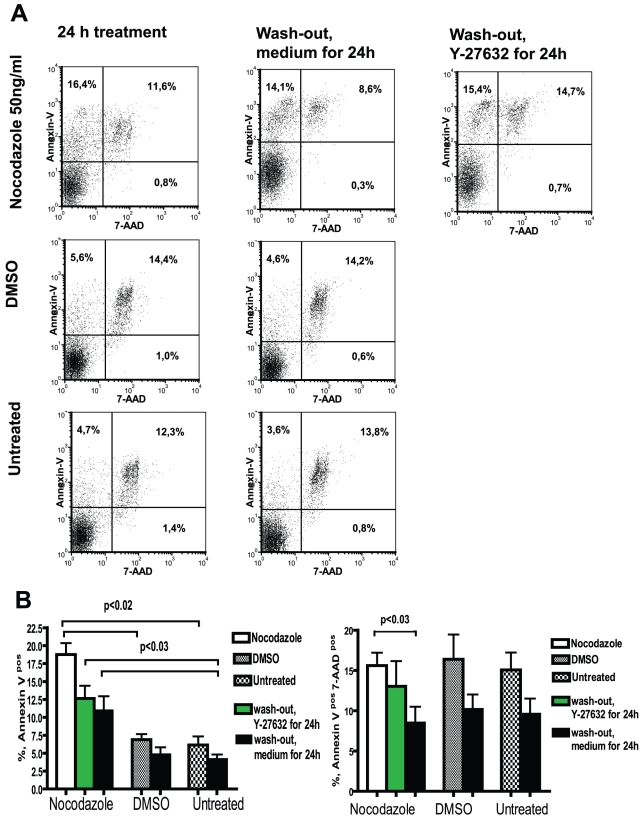
Apoptosis of hESC treated with nocodazole. hESC were exposed to nocodazole as described in Figure 3. Adherent cells were collected and stained with Annexin-V (PE conjugate) and 7-AAD. (A) Flow cytometric analysis of apoptotic cells (Annexin-V-positive cells) and necrotic and dead cells (Annexin-V-positive and 7-AAD-positive cells). (B) Summarising bar graphs of flow cytometric analysis of apoptotic cells and (B) necrotic and dead cells of four independent experiments.

hESC are especially sensitive to apoptosis, when dissociated into single cells and when colony formation has been disrupted. The number of apoptotic cells was estimated by staining living adherent cells with Annexin V (Figure 10). Apoptosis was more pronounced in nocodazole-treated cells and some apoptotic cells were detected in DMSO-treated or untreated cells. After release from nocodazole arrest, the rate of apoptosis decreased (Figure 10B), being still higher than in DMSO-treated or untreated cells. The addition of Y-27632 had no significant effect on the number of apoptotic cells. Nocodazole caused necrosis and death of cells to a similar extent as DMSO, but after the removal of nocodazole, the survival of cells improved (Figure 10B). The non-adherent cell population contained 30% of apoptotic cells and 70% of necrotic and dead cells, similarly in all studied cell sets. Among non-adherent cells the higher number of necrotic and dead cells was found in the G2/M phase after nocodazole treatment compared to DMSO-treated cells or untreated cells (data not shown).

**Figure 11:**
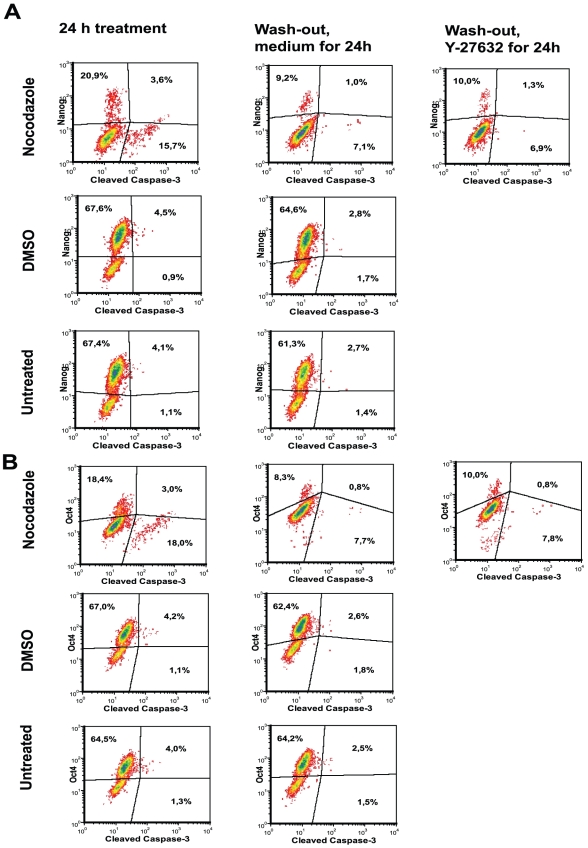
Expression of Nanog and Oct4 in apoptotic cells of hESC treated with nocodazole. hESC cells were treated with nocodazole as described in Figure 3. Cells were fixed and stained with anti-Nanog (PE), anti-Oct4 (Alexa Fluor 647) and anti-cleaved caspase-3 (detected with anti-rabbit IgG conjugated with Alexa 488) antibodies. (A) Expression of Nanog and (B) Oct4 in cleaved-caspase-3-positive cells. For analysis cellular debris and doublets were excluded. Results are shown as contour plots and are representative of two independent experiments.

To study whether the Nanog and Oct4 expressing cells were more prone to apoptosis, we investigated the presence of cleaved caspase-3, which characterises the late phase of apoptosis. Nocodazole treatment caused the cleavage of caspase-3 in cells; however, the majority of these cells were Nanog and Oct4 negative (Figure 11). In DMSO-treated and untreated cells the number of apoptotic cells with cleaved caspase-3 was low, but these cells were mostly Nanog and Oct4 positive. Thus, in normal culture conditions some Nanog-positive and Oct4-positive cells are more prone to apoptosis. We found a good correlation between the results of Annexin V staining and cleaved-caspase-3-positive cells (p<0.02).

**Figure 12:**
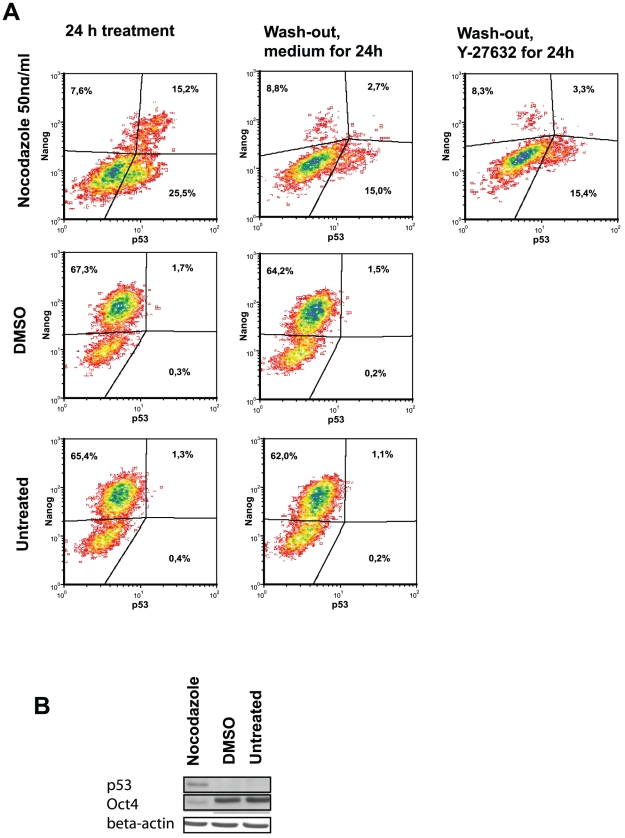
Protein p53 upregulation in hESC cells as result of nocodazole treatment. Fixed and permeabilised cells were stained with anti-p53 (Alexa Fluor 488) antibodies and anti-Nanog (PE) antibodies. (A) p53-positive cells and expression of Nanog in hESC. Results are shown as contour plots. For analysis cellular debris and doublets were excluded. (B) Cell lysates were characterised for presence of p53 and Oct4 by using Western blot assay. Detection of beta-actin was used as loading control.

As the nocodazole-treatment resulted in a decrease of Nanog and Oct4 expression in hESC, we next investigated whether the level of p53 was upregulated in these cells. p53 is known to directly regulate Nanog expression [31]. Nocodazole treatment increased the level of p53 in hESC (Figure 12A). There were two subpopulations of cells: p53^pos^Nanog^neg^ and p53^pos^Nanog^pos^ cell subpopulations. A higher number of cells were found in the subpopulation expressing p53 without Nanog expression in nocodazole-treated cells. The release from nocodazole resulted in a decrease of p53 expression, still being higher than in control DMSO-treated or untreated cells (less than 2% of p53-positive cells). The subpopulation p53^pos^Nanog^pos^ decreased significantly after the removal of nocodazole and its replacement with a fresh medium. There was no additional effect of Y-27632 on the expression of p53. The results of flow-cytometry analysis were confirmed by using the Western blot method, where the total adherent cell population was analysed (Figure 12B).

**Figure 13:**
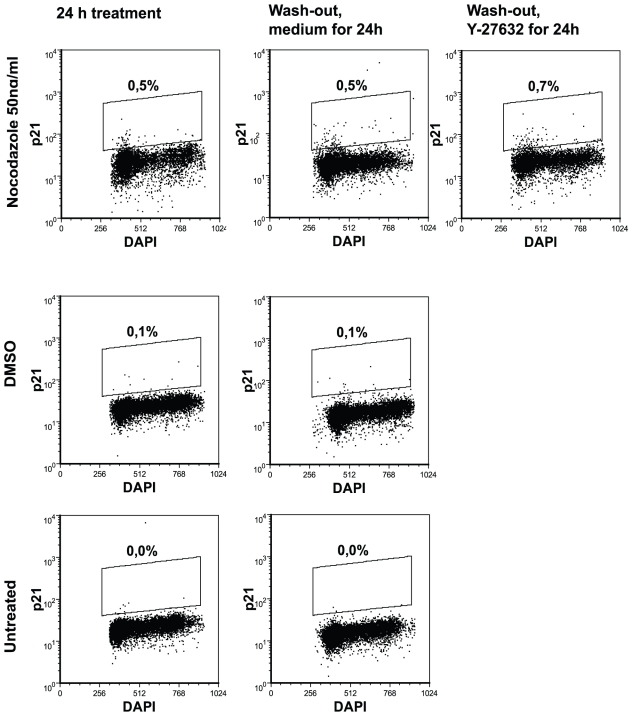
Detection of protein p21 ^WAF1/CIP1^ in nocodazole-treated cells. hESC were stained with anti-p21 ^WAF1/CIP1^ antibody (detected with anti-mouse IgG Alexa Fluor 647 conjugate) and DAPI. Results are shown as density plots and represent three independent experiments.

p53 is known to regulate various cellular processes including apoptosis, differentiation and genome integrity [32]. One of the p53 targets is p21 ^WAF1/CIP1^, the inhibitor of cell cycle phases in the G1/S and G2/M and this protein has also been reported to be upregulated as a response to nocodazole treatment in human non-small cell lung carcinoma cell-line A549 [33]. Although the expression of p21 ^WAF1/CIP1^ was rather low, we found a 5-fold increase in p21 ^WAF1/CIP1^ protein expression in nocodazole-treated cells compared to DMSO-treated or untreated cells. After nocodazole wash-out, the expression of p21 ^WAF1/CIP1^ was still detectable (Figure 13). Cells expressing p21 ^WAF1/CIP1^ were mostly in the G1 phase of the cell cycle (Figure 13).

## Discussion

In this study we report that nocodazole treatment of hESC causes a decrease in the expression of pluripotency markers Nanog and Oct4, while the level of p53 becomes up-regulated and accompanied by a high rate of apoptosis. After being released from nocodazole treatment, the cells still express very little Nanog and Oct4, although the cell cycle distribution becomes normal for ES cells. We also found that the expression of SSEA-3 and SSEA-4 was up-regulated in Nanog-expressing cells after release from nocodazole treatment.

Our findings are consistent with an earlier publication by Becker *et al* showing the loss of pluripotency factor Oct4 in nocodazole-synchronised cell cultures [34]. By using the flow cytometry method we found that the number of cells co-expressing both Nanog and Oct4 decreased significantly after nocodazole treatment. Cell cycle analysis revealed that the distribution of double-positive cells was higher in the G2/M phase, but lower in the S phase compared to treatment with DMSO or untreated cells. The majority of untreated or DMSO-treated cells were in the S phase, which is in agreement with an earlier report [35]. It has been shown that hESC cells are already pre-committed in mitosis to enter the next S phase in the absence of external stimuli [36]. Our results support this finding, since after the removal of nocodazole the distribution of cells in cell cycle phases changed towards an increased number of Nanog^pos^Oct4^pos^ cells in the S phase.

hESC undergo spontaneous apoptosis or anoikis (detachment-induced apoptosis) upon cellular detachment and dissociation. We noticed that intensive washing before adding a fresh growth medium improved survival and decreased apoptosis in untreated cells. Thus, the optimised culture conditions are essential to keep a spontaneous rate of apoptosis at a low level. In this study we used two different methods to estimate the rate of apoptosis. Annexin V staining was used to characterise the early stage of apoptosis, whereas a late stage of apoptosis was evaluated by detecting cleaved caspase-3 in fixed and permeabilised cells. The latter method allowed us to study the co-expression of Nanog and Oct4 in apoptotic cells. In nocodazole-treated cells cleaved caspase-3 was mostly detectable in Nanog and Oct4 negative cells. This was different in control DMSO-treated or untreated cells, where only a few cleaved caspase-3-positive cells co-expressed Nanog and Oct4. Thus, in normal (untreated) hESC culture, cells expressing both Nanog and Oct4 could be apoptotic. In nocodazole-treated cells it might be that Nanog and Oct4 expression was already downregulated in apoptotic cells. This can be caused by protein p53, since we found elevated levels of p53 in nocodazole-treated hESC. It has been shown earlier that p53 can directly bind to the promoter regions of Oct4 and Nanog, thus influencing protein expression levels [31]. In our experiments we could detect a population of cells co-expressing Nanog and p53 after nocodazole treatment, which has not been described earlier. This shows that the cells can express both pluripotency marker Nanog and p53 at the same time. Whether this is unique for human ESC should be clarified in further studies. However, p53 obviously has other functions in addition to downregulation of Nanog. For example, after release from nocodazole arrest some Nanog-positive cells still survived and the number of p53^pos^Nanog^neg^ cells decreased. It is therefore possible that the functional activity of p53 can be downregulated by the components in the fresh medium itself after the removal of nocodazole. Indeed, as recently shown, fibroblast growth factor 2 (FGF2) in a fresh medium is essential to up-regulate the level of Rem2 GTPase, which suppresses the transcription activity of p53 [37]. In untreated hESC the level of Rem2 GTPase is up-regulated and it is able to prevent protein degradation during DNA damage [37]. The removal of nocodazole block decreased apoptosis and p53 expression, but Nanog and Oct4 expression remained at the same low levels as during nocodazole arrest. The number of adherent cells and proliferating cells decreased, pointing to the possibility that damaged cells were not able to survive. Still, the majority of the survived adherent cells were undifferentiated expressing high levels of SSEA-4 and low levels of Gata-4.

Two different types of cell cycle arrest have been shown using another microtubule-stabilising agent taxol in various carcinoma cell lines [38], [39], [40]. Most of these cells were arrested in the pro-metaphase, which triggered rapid cell death independent of p53. The cells that could still pass mitosis were arrested in the subsequent G1 phase by a p53-dependent mechanism [40]. In our study the up-regulation of p21^WAF1/CIP1^ was only found in a few cells in the G1 phase, which could suggest that cells still passing mitosis were arrested in the G1 phase. Induction of p21 ^WAF1/CIP1^ has been related to the use of low concentrations of microtubule stabilising agents and may assist cell death and contribute to cell arrest in the G1 phase [41], [33]. It has been shown that in non-irradiated hESC cells (H1 cell line) p21 ^WAF1/CIP1^ mRNA levels are minimal and upon irradiation the level increases up to 300-fold, the p53/p21 ^WAF1/CIP1^ pathway gets activated and may respond to DNA damage and mediate cell cycle arrest by blocking DNA synthesis and allowing DNA repair or entry into apoptosis [42]. Since p21 ^WAF1/CIP1^ was detectable in very few cells it seems that the mechanism involving the p53/p21 ^WAF1/CIP1^ pathway might be less abundant in hESC treated with nocodazole.

We also characterised the expression of SSEA-3 and SSEA-4 antigens on the surface of hESC by using two different staining methods. We found that binding SSEA-3-specific monoclonal antibodies to glycosphingolipids on the cell surface had a better outcome in untreated and DMSO-treated cells, when living cells were stained. Incubation with Nanog-specific antibodies after fixing and permeabilisation of these stained cells allowed us to confirm that the majority of SSEA-3-positive cells also express Nanog. Thus, SSEA-3 antibodies can be used for sorting cells with high levels of pluripotency markers Nanog and Oct4. This is in agreement with a previous report showing that cells expressing SSEA-3 differ from the SSEA-3 negative cell population by their expression of Nanog and Oct [43]. SSEA-4-specific monoclonal antibodies binding at the cells previously fixed and permeabilised resulted in several populations, while the expression of Nanog was detected in the population of SSEA-4^pos^ cells with high mean fluorescent intensity. This finding is in line with the previous report showing that SSEA-4 antibodies bind preferably to GL7 epitopes in glycosphingolipids, which is the longest carbohydrate chain among the glycolipids detected in hESC, and also to glycolipids with shorter carbohydrate chains [29]. The latter ones are frequently not detectable unless the cell membrane is disrupted [44]. This could explain the different binding profile of SSEA-4 antibodies to fixed and permeabilised cells, since the epitopes unreachable in living cells can be easily recognised after disruption of the cell membrane. After the release from nocodazole arrest the expression of both SSEA-3 and SSEA-4 increased, probably reflecting the changes in cell surface glycolipids as the first event.

Flow cytometry analysis applied in this study allowed us to characterise a single cell for several parameters at the same time, which is a novel approach for characterisation of hESC. This powerful method gives valuable results in understanding the propagation of the cell cycle of hESC. However, for hESC the flow cytometric analysis is more complicated compared to analysis of other cell lines due to high non-specific binding, autofluorescence and a limited number of antibodies with high specificity. Usually a single parameter flow cytometric analysis has been used and the expression level of studied proteins has been reported to be higher (70–99%) than in our study. This difference could be explained by different antibodies used in various studies and by different population selection criteria. In this study we used selection of DAPI-positive cells, which were further analysed for protein expression. In addition to that, we used at least two different parameters (antigens) and applied density plots instead of histograms, which can easily over-estimate the positive population when there is no clear borderline between positive and negative populations. Altogether, the optimised conditions for cell fixation and antibody staining resulted in stricter population selection in our study. Still, our results depend on the specificity and avidity of the particular antibodies used in the experiment.

Nocodazole treatment is widely used to arrest cells in mitosis. Here we show that the majority of cells in the G2/M phase possessed phosphorylated H3 and obviously were arrested in the M phase. Recently Teperek-Tkacz with co-workers have shown that during the first and second cell cycles of mouse embryo the H3 phosphorylation-dephosphorylation at Ser10 differs from the one observed in somatic cells by prolonged phosphorylation following anaphase [45]. This was due to sustained kinase and not lower phosphatase activity. *De novo* phophorylation of H3 at Ser10 started at the end of the S phase and was detectable during the G2 and the whole M phase including ana/telophase. Dephosphorylation of H3 at Ser10 started not earlier than at the G1 phase. Phosphorylated H3 at Ser10 was present in pronuclei and blastomere nuclei in early G1 phase, but not during early or middle S phase [45]. In somatic cells, phosphorylation of H3 at Ser10 appears first in late G2-interphase cells. Subsequently, the phosphorylation of H3 at Ser10 spreads along the chromosomes, it is completed at the prophase and is still evident in the metaphase plate [46]. Using a low concentration of nocodazole we detected phosphorylation of H3 at Ser10 in the G2/M and G1 phases. Why in untreated and DMSO-treated cells the population in the G1 phase was not recognised can be explained by the low number of cells with phosphorylated H3. In addition to Ser10 phosphorylation we also evaluated the phosphorylation of Ser28 of H3. The results were comparable with those for phosphorylated Ser10. This is in agreement with previous reports, where similar distribution of H3 phosphorylation at Ser28 and Ser10 was observed, however, the initiation of phosphorylations differed [47]. Our experimental set-up could not give additional information to this question.

Nocodazole treatment increases cell detachment due to the destabilisation of microtubules. We therefore used ROCK-2 inhibitor Y-27632 after nocodazole wash-out in order to improve the survival, cell-cell interactions and cell attachment to Matrigel®. After 24 hours of treatment with Y-27632 we detected the formation of small colonies not visible on control plates without the ROCK-2 inhibitor. The number of Nanog^pos^Oct^pos^, however, remained at the same low level indicating that the expression of Nanog and Oct4 could not be restored along with normalisation of the cell cycle distribution similarly when Y-27632 was absent. The addition of Y-27632 could not decrease the rate of apoptosis, which is in contradiction with an earlier study, where Y-27632 markedly decreased the rate of dissociation-induced apoptosis of hESC [48]. We found that the levels of p53 were still elevated after nocodazole release and the addition of a fresh medium with Y-27632. It might be that in the apoptotic cells signalling pathways were already activated and the presence of Y-27632 could not have any effect. We observed that in both DMSO-treated cells and untreated cells the addition of Y-27632 increased the proliferation of cells. It seems that Y-27632 preferably and selectively influences the untreated (normal) cells, decreasing the survival of manipulated cells.

The results of this study underline that in context of hESC it is beneficial to have detailed characterisation of cells at the single cell level whenever the cells have been manipulated. This *in vitro* screening could be a valuable tool for estimating the effect of medicinal agents or potential medicinal agents on the hESC *in vivo*.

## Supporting Information

Figure S1 **Effect of ROCK-2 inhibitor Y-27632 on DMSO-treated and untreated cells.** On day 5 after passaging of cells, hESC were treated with DMSO (0.01%, same amount of DMSO was added to cells with nocodazole solution in DMSO) or left untreated. After 24 h cells were washed with fresh medium and medium containing Y-27632 (20 µM) was added for subsequent 24 h. Results were compared with hESC treated with fresh medium for 24 h instead of Y-27632 containing medium. Cells were stained as described in Figure 2 and in Figure 7. (A) Cell cycle profile of hESC. (B) Expression of Nanog and Oct4 and (C) expression of SSEA-4 and Nanog in hESC. Results are shown as contour plots and represent two independent experiments.
(TIF)
