# Supplementary figures and images for: Nocodazole Treatment Decreases Expression of Pluripotency Markers Nanog and Oct4 in Human Embryonic Stem Cells
Source: PLoS One. 2011 Apr 29;6(4):e19114. doi: 10.1371/journal.pone.0019114 (PMC3084750; doi:10.1371/journal.pone.0019114)

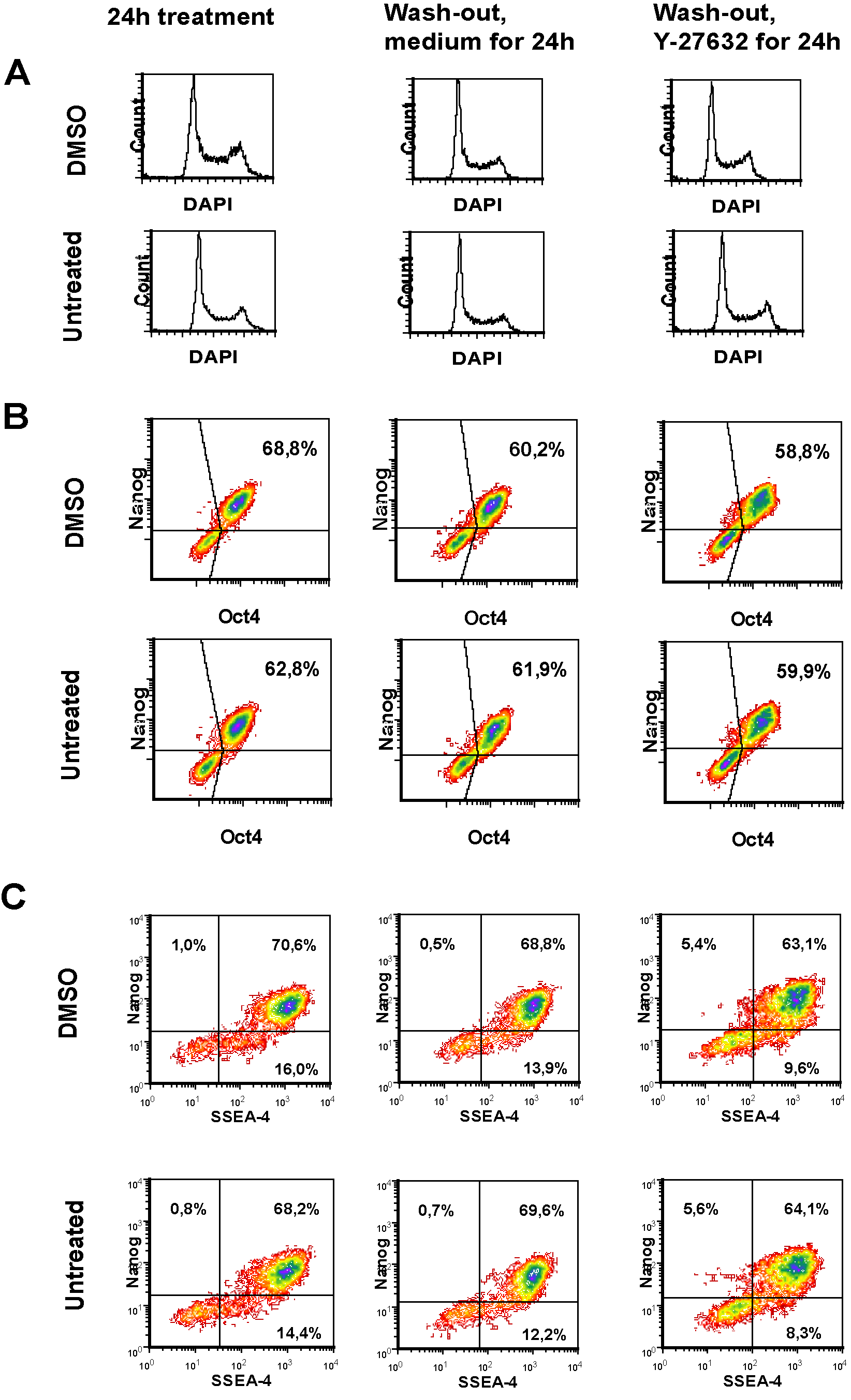

Supplement: Figure S1 — Effect of ROCK-2 inhibitor Y-27632 on DMSO-treated and untreated cells. On day 5 after passaging of cells, hESC were treated with DMSO (0.01%, same amount of DMSO was added to cells with nocodazole solution in DMSO) or left untreated. After 24 h cells were washed with fresh medium and medium containing Y-27632 (20 µM) was added for subsequent 24 h. Results were compared with hESC treated with fresh medium for 24 h instead of Y-27632 containing medium. Cells were stained as described in Figure 2 and in Figure 7. (A) Cell cycle profile of hESC. (B) Expression of Nanog and Oct4 and (C) expression of SSEA-4 and Nanog in hESC. Results are shown as contour plots and represent two independent experiments. (TIF) [file pone.0019114.s001.tif]
